# ITGA6+ Human Testicular Cell Populations Acquire a Mesenchymal Rather than Germ Cell Transcriptional Signature during Long-Term Culture

**DOI:** 10.3390/ijms21218269

**Published:** 2020-11-04

**Authors:** Robert B. Struijk, Callista L. Mulder, Saskia K. M. van Daalen, Cindy M. de Winter-Korver, Aldo Jongejan, Sjoerd Repping, Ans M. M. van Pelt

**Affiliations:** 1 Reproductive Biology Laboratory, Center for Reproductive Medicine, Amsterdam UMC, Amsterdam Reproduction & Development Research Institute, University of Amsterdam, 1105 AZ Amsterdam, The Netherlands; r.b.struijk@amsterdamumc.nl (R.B.S.); C.L.Mulder@amsterdamumc.nl (C.L.M.); s.k.vandaalen@amsterdamumc.nl (S.K.M.v.D.); c.m.korver@amsterdamumc.nl (C.M.d.W.-K.); 2 Department of Epidemiology & Data Science, Amsterdam UMC, Amsterdam Public Health Research Institute, University of Amsterdam, 1105 AZ Amsterdam, The Netherlands; a.jongejan@amsterdamumc.nl; 3 Amsterdam UMC, University of Amsterdam, 1105 AZ Amsterdam, The Netherlands; S.Repping@amsterdamumc.nl

**Keywords:** fertility preservation, spermatogonia, stem cells, bulk transcriptome, germ cells, cell culture, cell type deconvolution

## Abstract

Autologous spermatogonial stem cell transplantation is an experimental technique aimed at restoring fertility in infertile men. Although effective in animal models, in vitro propagation of human spermatogonia prior to transplantation has proven to be difficult. A major limiting factor is endogenous somatic testicular cell overgrowth during long-term culture. This makes the culture both inefficient and necessitates highly specific cell sorting strategies in order to enrich cultured germ cell fractions prior to transplantation. Here, we employed RNA-Seq to determine cell type composition in sorted integrin alpha-6 (ITGA6+) primary human testicular cells (*n* = 4 donors) cultured for up to two months, using differential gene expression and cell deconvolution analyses. Our data and analyses reveal that long-term cultured ITGA6+ testicular cells are composed mainly of cells expressing markers of peritubular myoid cells, (progenitor) Leydig cells, fibroblasts and mesenchymal stromal cells and only a limited percentage of spermatogonial cells as compared to their uncultured counterparts. These findings provide valuable insights into the cell type composition of cultured human ITGA6+ testicular cells during in vitro propagation and may serve as a basis for optimizing future cell sorting strategies as well as optimizing the current human testicular cell culture system for clinical use.

## 1. Introduction

Spermatogonial stem cells (SSC) are the progenitor cells of male gametes, developing to spermatozoa in the seminiferous epithelium of the testis through a specialized process called spermatogenesis. Since SSCs are sensitive to damage originating from common gonadotoxic therapies, including chemo- and radiotherapy [1,2,3], survivors of cancer or hematological diseases are predisposed to develop infertility after they have been cured [4]. This is especially devastating for prepubertal patients that cannot benefit from sperm cryopreservation, in case they received gonadotoxic treatment before spermatogenesis has been initiated.

Although mature sperm cells are absent in young patients, a functional pool of SSCs is present from birth. Autotransplantation of SSCs into the testis has been suggested as a novel fertility restoration approach to restore sperm production in sterile male survivors of cancer or hematological diseases during childhood [5,6,7,8]. More specifically, this technique involves isolation of SSCs from a cryopreserved testicular biopsy that was taken prior to treatment, expansion of SSCs in vitro and autotransplantation of SSCs (SSCT) after successful treatment when sterility has manifested in the patient [9]. Upon recolonization in the testicular niche, the transplanted in vitro propagated SSCs are then capable of starting the process of life long spermatogenesis, ultimately allowing for natural conception to fulfill their child wish.

The colonization success rate of SSCT is dependent on and positively correlated with the number of transplanted SSCs [10,11]. As such, efficient in vitro expansion of an initial low number of SSCs isolated from a small prepubertal testicular biopsy is a critical aspect of this therapy [12,13]. The first report of efficient in vitro long term SSC propagation was reported in the mouse and since then researchers have shown efficient in vitro proliferation of SSCs of multiple non-human species [14,15,16,17,18,19,20]. Over a decade ago, the first culture protocol for human testicular cells with the aim of expanding the number of human SSCs in culture was developed [21,22], and subsequently, other groups published on in vitro propagation of human SSCs as well [23,24,25].

The development of a human SSC culture system that is comparable to other animal models in terms of efficiency has proven to be more challenging. Based on experience from animal SSC culture systems, it is widely accepted that SSCs are difficult to propagate without support from somatic feeder cells. In mouse culture systems, endogenous somatic cells are replaced by a layer of inactivated embryonic mouse fibroblasts that function as feeder cells with the purpose of supporting efficient SSC proliferation and preventing somatic cell overgrowth within the culture environment [16,17,26]. Since the use of animal-derived products should be avoided in clinical therapeutic approaches in humans, a feeder layer of endogenous testicular somatic cells obtained from a patient’s own testicular tissue is the preferred approach for establishing and expanding SSCs in vitro. A limiting factor in current human SSC culture models is that endogenous testicular somatic cells drastically overgrow the culture when they are not reduced in number during culture, thereby diluting the relative number of SSCs in long-term testicular cultures and subsequent low transplantation efficiency rates [27,28,29]. The diminished germ cell percentage and the presence of a considerable somatic component in human testicular cell cultures thus complicates and limits the clinical applicability of SSCT using currently available methodologies.

To circumvent the issue of low SSC percentages in testicular cell cultures available for transplantation, we and other groups have employed magnetic-activated cell sorting (MACS)- or fluorescence-activated cell sorting (FACS)-based cell sorting strategies using published spermatogonial markers to enrich the heterogeneous cultured testicular cell population for functional spermatogonial cells [30,31]. As an example, the laminin receptor protein integrin alpha-6 (CD49f/ITGA6) has been suggested as an in vivo spermatogonial cell surface marker in both rodents and humans [31,32,33]. MACS sorting of cultured human testicular cells for ITGA6+ cells prior to transplantation resulted in the highest measured (7.1-fold) increase in the number of SSC derived colonies after xenotransplantation to recipient mouse testis, as compared to transplantation of unsorted cultured testicular cells or using other markers such as GPR125^+^/HLA^−^ cells [30].

Despite the application potential for SSC enrichment using ITGA6+ cell sorting, testicular expression of the ITGA6 protein is not confined to spermatogonial stem cells and it is therefore to be expected that ITGA6+ cell fractions from long-term cultures will include multiple cells types such as mesenchymal stromal cells [34], or other somatic cell types native to the in vivo testis. Due to its potential application in clinical SSC transplantation, it is crucial to identify the different cell populations present within the ITGA6+ subpopulation during long term culture. Determination of the cell type composition in the ITGA6+ subpopulation of long-term cultured human testicular cell cultures may serve both to determine whether additional (negative) sorting for certain somatic cell type markers is required, as well as provide further insights into factors that can be adapted in the current human SSC culture system to decrease growth rates of overgrowing somatic cell types, thus increasing the yield of germ cells usable for transplantation purposes.

Here we set out to determine the cell type composition of cultured ITGA6+ sorted human primary testicular cells at the initiation of culture and throughout in vitro propagation up to two months (including six time points) using RNA-Seq, followed by extensive differential gene expression analyses, gene set enrichment analysis and cell type deconvolution.

## 2. Results

### 2.1. Derivation of ITGA6+ Testicular Cell Fractions from Mixed Testicular Cell Cultures

**Figure 1:**
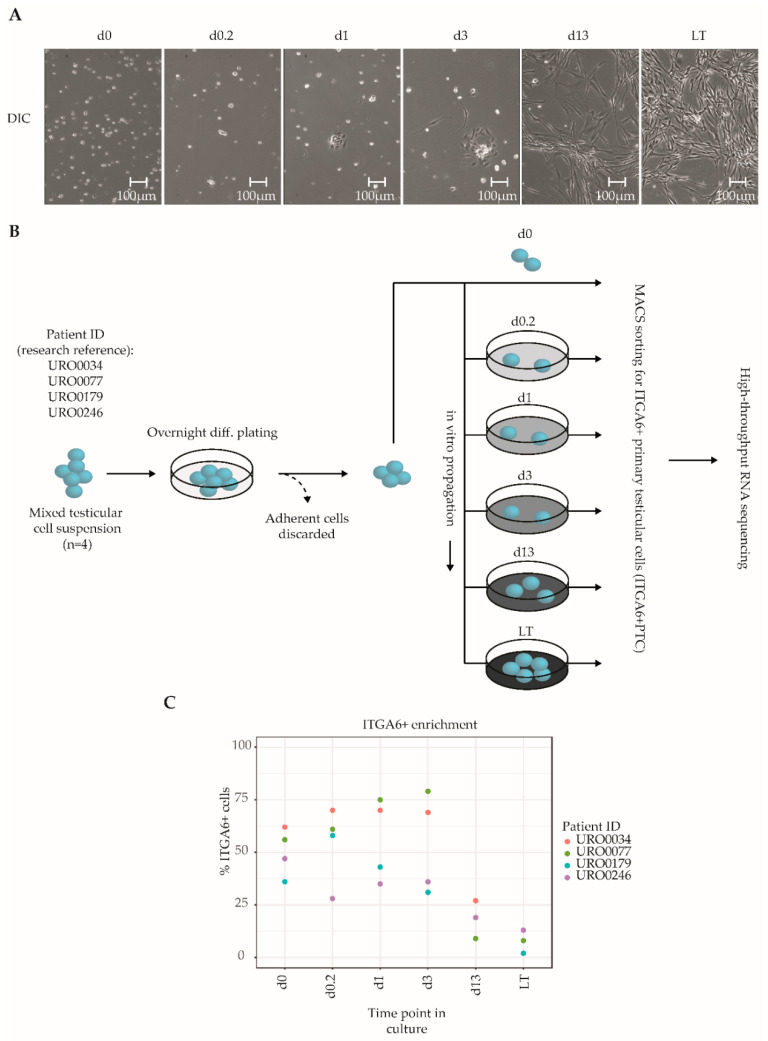
Overview of the culture protocol used to isolate ITGA6 + PTCs from human adult testis. (**A**) Differential interference contrast microscopy images of unsorted testicular cell fractions at different time points in culture, consisting of ingrowing spindle-shaped somatic cells and round germ cells. (**B**) Mixed testicular cell suspensions were obtained from cryopreserved testicular biopsies (*n* = 4) using a two-step enzymatic digestion protocol and either directly sorted for ITGA6+ cells or first put into culture for the designated duration of time and then sorted for ITGA6+ cells. In total, six culture time points were analyzed by high-throughput RNA sequencing: 0 h (d0), 4 h (d0.2), 24 h (d1), 72 h (d3), two weeks (d13) and two months (LT, long-term). (**C**) Scatterplot displaying the percentage of ITGA6 + PTCs as compared to the total population obtained through MACS sorting for each donor at each time point.

Primary testicular cells (PTC) were isolated from cryopreserved testicular tissue biopsies (*n* = 4 different donors) and propagated in vitro for up to two months, which is the postulated culture duration required to obtain sufficient cell numbers for successful transplantation based on human to mouse xenotransplantation experiments [21,22]. A total of six culture time points were analyzed in the current study: At the onset of culture to get the cells settled day 0 (d0), after 4 h (d0.2), 24 h (d1), to an almost complete monolayer before first passage at 72 h (d3) and then at two weeks (d13) and two months after 5–6 passages (LT, long-term). The chosen time points reflect distinct morphological changes of the PTC culture. During culture, we observed a notable transition from mostly floating, round cells at the onset of culture to a pronounced monolayer of attached spindle-shaped cells from day 13 onwards, with a smaller population of round germ cells adhering to the monolayer (Figure 1A, Video S1), consistent with previous studies that have reported similar transitions [21,22,29,35]. Throughout culture, PTCs were enriched for SSCs through MACS sorting for ITGA6+ cells, resulting in SSC-enriched PTC fractions (hereafter termed ITGA6 + PTC) obtained at six culture time points from four unique testicular tissue donors (total of 24 fractions) (Figure 1B). Consistent with the aforementioned reduction in the ratio of round germ cells as compared to adherent spindle-shaped somatic cells, we detected a significant reduction in the percentage of ITGA6 + PTCs as compared to unsorted PTC with increasing culture time (mean ± SD; 60% ± 16% at day 0 to 6% ± 5% after long-term culture) (Figure 1C).

### 2.2. Long-Term In Vitro Propagation of ITGA6 + PTCs Is Correlated with Distinct Transcriptional Changes

**Figure 2:**
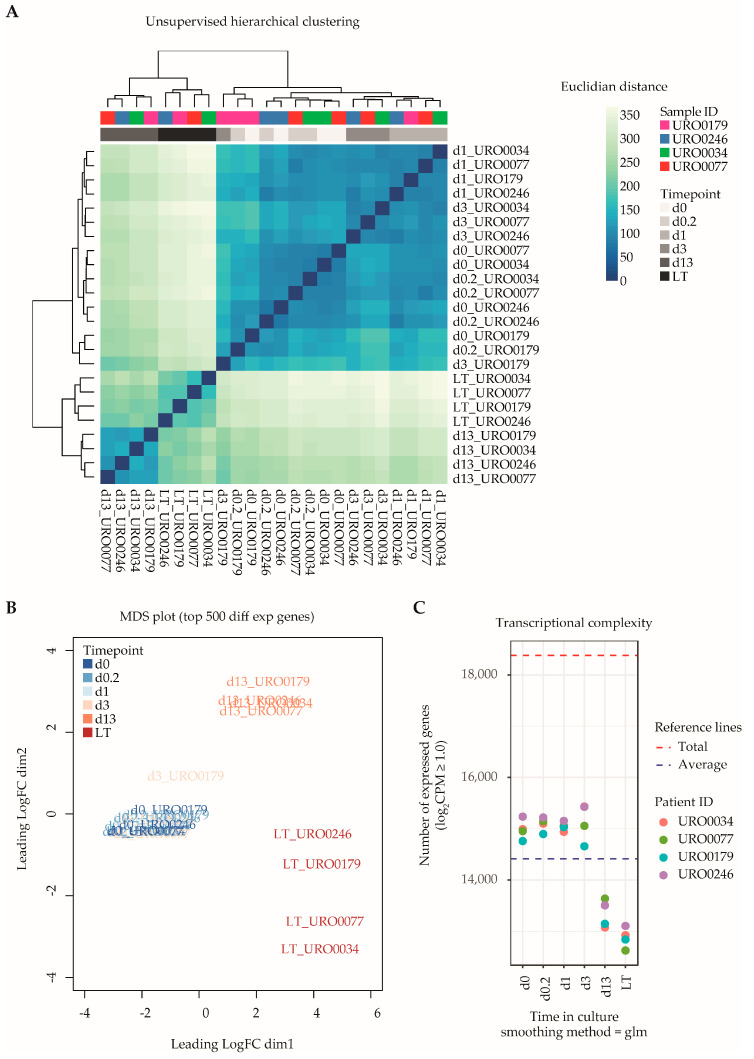
Global transcriptomic analyses of ITGA6 + PTCs during culture. (**A**) Unsupervised hierarchical clustering analysis shows clear separation of ITGA6 + PTCs into three distinct groups, corresponding to time in culture. (**B**) MDS plot displaying distance between samples based on the top 500 most variable genes. (**C**) Transcriptional complexity as measured by the number of unique gene entries with a log2CPM (counts-per-million) value of ≥1.0. Both d13 and LT time points displayed significantly lower transcriptional complexity as compared to d0 (ANOVA *p*-value 1.25 × 10^−11^, followed by post hoc analyses using Tukey’s HSD (honest significant differences) tests with a cutoff value of *p*-value ≤ 0.01).

Next, we generated transcriptional data sets of ITGA6 + PTCs throughout culture by RNA-Seq, spanning a total of 18,380 unique RefSeq-annotated gene identifiers after data normalization and filtering. Unsupervised hierarchical clustering analysis based on total transcriptomic profile revealed separation of samples into three distinct groups according to time in culture (Figure 2A), namely d0–d3, d13 and LT-ITGA6 + PTCs. This was further substantiated by measuring the distance between samples based on the top 500 most variable genes (Figure 2B). The transcriptional changes associated with in vitro propagation did not appear to occur linearly, since d13-ITGA6+PTCs clustered further away from d0-ITGA6 + PTCs as compared to LT-ITGA6 + PTCs. Following the observed clustering pattern, differential gene expression analysis revealed a substantial increase in the number of differentially expressed genes (DEG) when comparing successive ITGA6 + PTCs per patient during culture (Figure S1). In addition to increased differential expression in d13-ITGA6 + PTCs and LT-ITGA6 + PTCs, a significant decrease in transcriptional complexity was observed with increasing culture time (*p* = 1.25 × 10^−11^, one-way ANOVA test). Compared to 14,985 total transcripts with a logarithmic counts-per-million (log2CPM) score of ≥1 at d0 in culture, the number of expressed transcripts was significantly reduced in d13 (13,345 transcripts, *p* ≤ 0.01, Tukey HSD test) and LT (12,772 transcripts, *p* ≤ 0.01, Tukey HSD test) cultures (Figure 2C).

### 2.3. Long-Term Cultured ITGA6 + PTCs Possess Mesenchymal and Fibroblast Related Gene Expression Signatures

To further investigate the implication of the observed differences in gene expression occurring during culture, gene set enrichment analysis (GSEA) was employed to find sets of genes that show concordant expression with predefined biological signatures recorded in the curated MSigDB database. When comparing d0-ITGA6 + PTCs with LT-ITGA6 + PTCs, none of the interrogated gene sets were enriched when applying a correction for multiple testing (FDR cutoff ≤ 0.01), but a number of data sets showed weak association when applying an unadjusted *p*-value ≤ 0.05 (Table 1).

**Figure 3:**
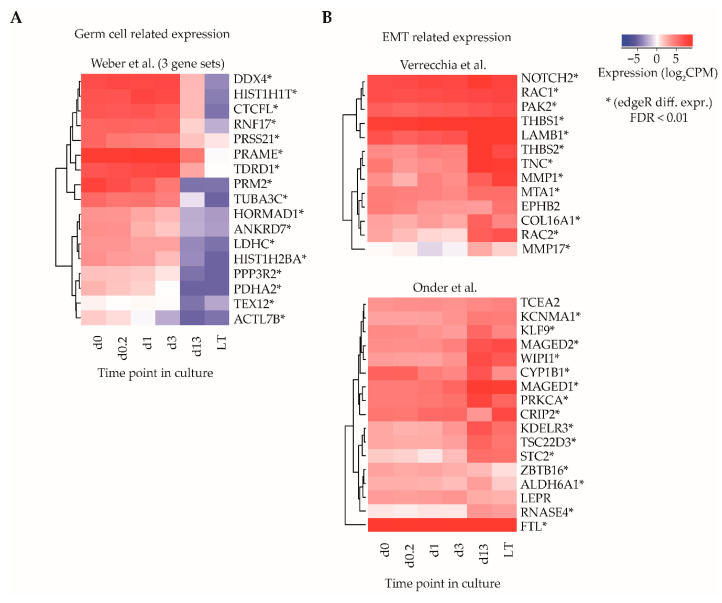
Gene set enrichment analysis for identification of sets of functionally related genes. (**A**) Average log2CPM of gene identifiers in three gene sets described by Weber et al., correlating to transcriptional differences between germ cells and fibroblasts and (**B**) two gene sets described by Verrecchia et al. and Onder et al., relating to epithelial-to-mesenchymal transition (EMT) transcriptional signatures. Asterisks indicate whether the gene was differentially expressed (edgeR, adjusted *p*-value cut off (FDR) ≤ 0.01).

Among the top 20 gene sets in the category with weak statistical association, three gene sets related to a study of Weber and colleagues, who described the dynamics of promoter DNA methylation in lung fibroblasts as compared to mature sperm cells [36]. In their paper, gene sets were identified that show high methylation at promoter regions in fibroblasts (transcriptionally silenced gene) and low methylation in sperm (transcriptionally active gene), and it was postulated that these gene sets might be involved in germ line development and/or maturation. The set of 17 genes listed by Weber et al. were all significantly downregulated when comparing d0-ITGA6 + PTCs with LT-ITGA6 + PTCs (adjusted *p*-value < 0.01, edgeR differential gene expression analysis) (Figure 3A).

Several other gene sets with weak statistical association were involved in the epithelial-to-mesenchymal transition (EMT) signature [37], as well as integrin 1 and 5 pathways in one of the clusters in the gene set of the study of Verrecchia et al. [38], response to TGFB1 [39] and CDH1 regulation in one of the clusters in the gene set of the study of Onder and colleagues [40]. A total of 27/30 genes in the sets associated with EMT showed significant upregulation (adjusted *p*-value < 0.01, edgeR differential gene expression analysis) in LT-ITGA6 + PTCs as compared to d0-ITGA6 + PTCs (Figure 3B).

Taken together, these data may provide insights in gene expression changes and thereby potential proportion of germ cells and somatic cells in LT-ITGA6 + PTCs compared to d0-ITGA6 + PTCs.

### 2.4. Analysis of Established In Vivo and In Vitro Testicular Cell Type Marker Genes

**Figure 4:**
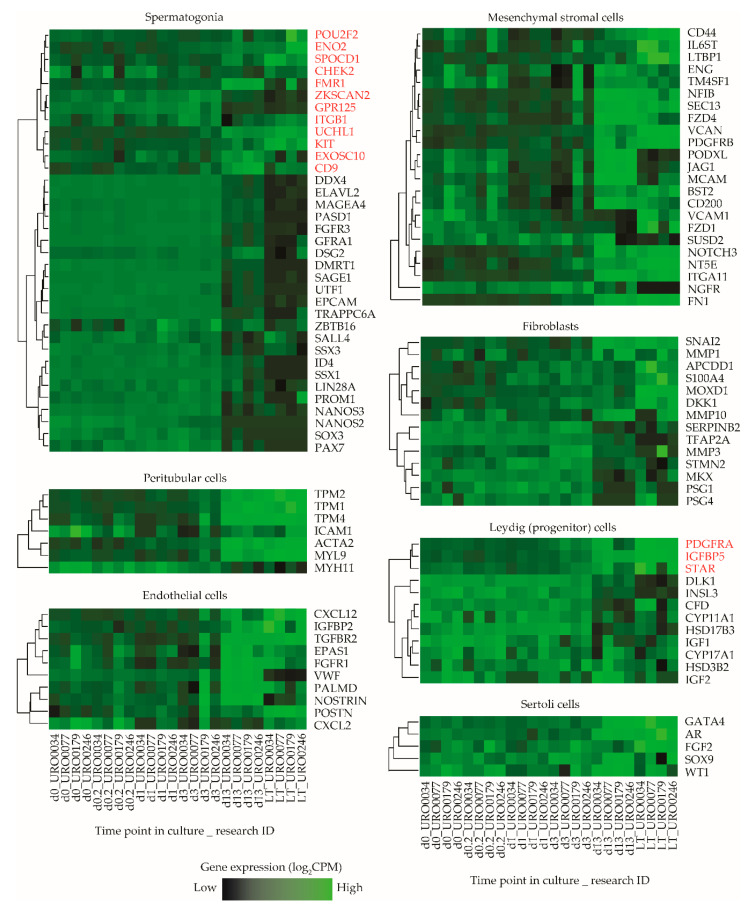
Expression levels of selected marker genes in ITGA6 + PTCs during culture. Heatmap of expression patterns in ITGA6 + PTCs during culture of in vivo cell type specific marker genes for spermatogonia, peritubular myoid cells, endothelial cells, mesenchymal stromal cells, fibroblasts, (progenitor) Leydig cells and Sertoli cells. Genes written in red were designated as outliers based on hierarchical clustering and subsequently excluded from downstream cell decomposition analyses.

To obtain a more robust estimate of the ratio in which particular cell types of both germ cell and somatic cell lineages might be present in ITGA6 + PTC cultures, we next analyzed a list of cell type specific marker genes associated with several predominant cell types found in the seminiferous epithelium and surrounding interstitium within the in vivo testis or in vitro in long term PTC cultures (spermatogonia, Sertoli cells, (progenitor) Leydig cells, peritubular myoid cells, fibroblasts and mesenchymal stromal cells) (Table S1) [25,34,41,42,43,44,45,46,47,48,49,50,51]. By carefully reviewing the literature, we identified a total of 106 genes as selective biomarkers for these cell types and analyzed expression levels of these genes at various time points during culture in more detail (Figure S2). Remarkably, while gene expression levels follow a consistent pattern of all selected genes for most cell types, this was not the case for spermatogonial and (progenitor) Leydig cell marker genes, which clustered into two separate subgroups of markers characterized by a major set of genes with concurrent gene expression levels and a smaller set of outlier genes based on their expression during culture (Figure 4). Of the analyzed spermatogonial markers, 12/35 genes (*POU2F2*, *ENO2*, *SPOCD1*, *CHEK2*, *FMR1*, *ZKSCAN2*, *GPR125*, *ITGB1*, *UCHL1*, *KIT*, *EXOSC10*, *CD9*) displayed expression values inconsistent with the remaining 23/35 markers and were upregulated rather than downregulated during culture. Similarly, 3/12 (progenitor) Leydig cell markers (*PDGFRA1*, *IGFBP5*, *STAR*) were upregulated during culture, while the remaining 9/12 showed a heterogeneous expression pattern between samples (Figure 4).

Next, a k-means clustering analysis was carried out to group all expressed genes in the sample libraries into six subclusters based on their expression pattern and overlaid the defined marker gene list in Table S1 onto the subclusters in order to determine whether the outlier spermatogonial and (progenitor) Leydig cell markers that showed inconsistent expression clustered with markers of other cell type (s) instead. This analysis revealed that most outlier spermatogonia-related genes group with markers of all analyzed testicular somatic cell types (subcluster 1, 3, 4 and 6) and, similarly, outlier (progenitor) Leydig cell-related genes group with markers of all other cell types (subcluster 1) (Figure S3).

### 2.5. Cell Decomposition Analysis Points towards a Decrease in the Proportion of Spermatogonia in ITGA6 + PTCs over Culture Time Accompanied by an Increase in Mesenchymal Cells

**Figure 5:**
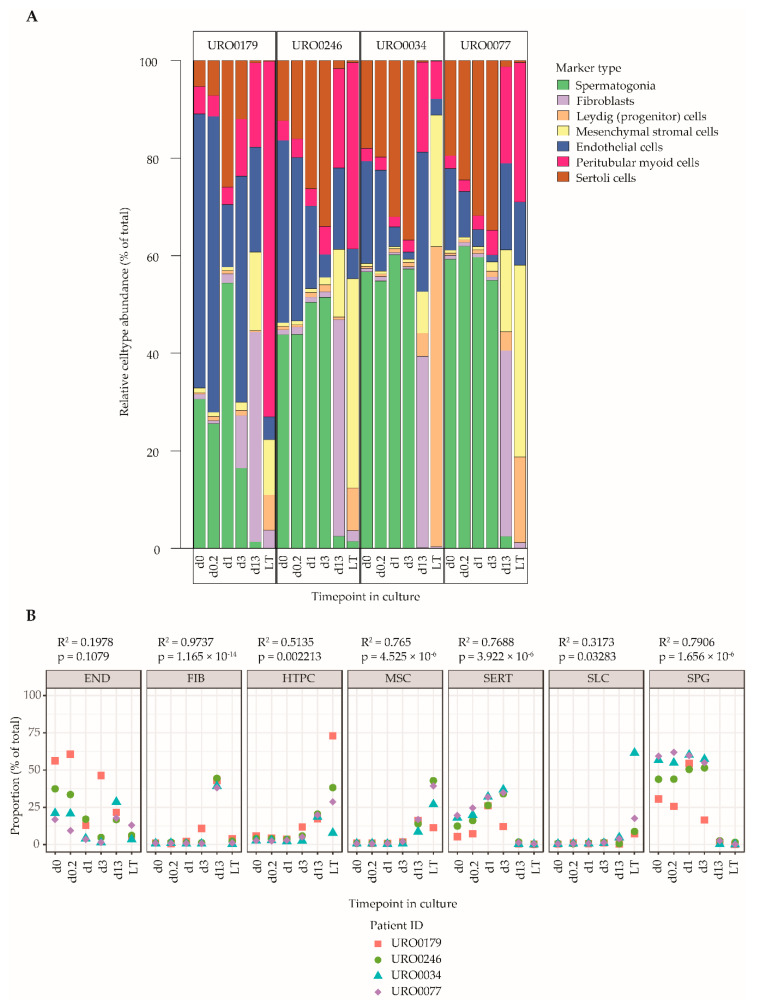
Cell decomposition analyses of ITGA6 + PTCs during culture using Cell Mix. (**A**) Relative abundance of cell types in the ITGA6+PTC during culture based on cell type specific gene expression patterns (see Table S1 and Figure 4). (**B**) Scatterplot of abundance of each cell type over time in culture in the ITGA6 + PTC for each donor. A linear regression was carried out to detect cell types that show significant differences in proportion with increasing culture time (linear regression; goodness-of-fit is measured by *R*-squared values, *p*-values indicate *F*-statistic, significance cut off at *p* ≤ 0.01).

Finally, a cell type deconvolution analysis using CellMix [52], was performed to estimate cell type proportions in ITGA6 + PTCs during culture using the marker genes defined in Table S1 and excluding outlier genes as identified in Figure 4. In line with the differential gene expression analyses and gene set enrichment data, this analysis revealed an altered cell type composition profile in d13-ITGA6 + PTCs and LT-ITGA6 + PTCs as compared to d0-ITGA6 + PTCs, characterized by a reduction in the proportion of spermatogonia in favor of somatic cell types (Figure 5A). Specifically, in d0-ITGA6 + PTCs, the predominant expression patterns correspond mostly to cells of spermatogonial and endothelial lineages, and to a lesser extent, peritubular myoid cells and Sertoli cells. In contrast, the expression profile of the spermatogonial and endothelial markers were both greatly decreased in d13-ITGA6 + PTCs and LT-ITGA6 + PTCs while the signature of mesenchymal stromal cells and peritubular myoid cells increases significantly (Figure 5B). Fibroblasts were detectable in d13-ITGA6 + PTCs but not in LT-ITGA6 + PTCs. Progenitor Leydig cells were almost undetectable in d0-ITGA6 + PTCs up to d13-ITGA6 + PTCs and showed variable abundance between LT-ITGA6 + PTC samples. The expression profile associated with Sertoli cells initially increased at the early time points (d0.2, d1 and d3), followed by a steep decline in d13-ITGA6 + PTCs and LT-ITGA6 + PTCs. Despite some variation between patient samples, the general picture pointing towards a decrease in spermatogonial percentages and a more mesenchymal cell type signature is evident in all patient samples.

## 3. Discussion

In the current study, we set out to determine the cell type composition of human ITGA6+ enriched primary testicular cells (ITGA6 + PTC) over the course of up to two months propagation culture by employing RNA-Seq. Based on cell type decomposition analyses over time during culture, ITGA6 + PTCs appear to have a distinct cellular composition that is characterized by cell populations with a decreasing spermatogonial-related gene expression profile and an increasing gene expression profile related to cells of mesenchymal, fibroblast and peritubular myoid origins.

In the primary testicular cell cultures examined here, a notable macroscopic shift from mostly non-adherent round cells to a pronounced monolayer of cells morphologically resembling spindle shaped fibroblast-like cells was observed, as well as a reduction in the percentage of ITGA6 + PTCs in long-term PTC cultures. In terms of transcriptional profile, transcriptional complexity was decreased significantly in long-term cultured ITGA6 + PTCs, which is in line with a decrease in the percentage of spermatogonia as spermatogonia possess high degrees of transcriptional complexity (i.e., number of unique transcripts expressed). This high degree of transcriptional complexity has been suggested to be a hallmark of undifferentiated germ cells [53], as it allows for complex regulation of cellular states and flexibility in terms of differentiation.

Our findings of a reduced percentage of spermatogonia after long term culture are in line with previous studies [54,55]. Using a similar culture system as in the current study, Medrano et al. [56] also reported a diminished proportion of putative SSCs cell clusters and an increase of spindle-shaped cells when cultured for 14 days. Interestingly, RT-PCR revealed an upregulated expression of genes expressed in somatic cells such as ACTA2 and STAR between culture day 7 and 14 similar as in our current study. Interestingly, in our data, we found an increase in expression profiles associated with Sertoli cells early in culture, followed by a sharp decrease in later time points. It is known that Sertoli cells resume proliferation in short term cultures [57], consistent with our results. A decrease of Sertoli cells in long term PTC culture was also reported earlier by Baert et al., 2015 [29], confirming our results. Sertoli cells play a critical role in spermatogonial self-renewal in vivo by providing among others GDNF [58], a growth factor that is substituted by recombinant forms in vitro [16,21]. One could argue that the decline in spermatogonial could be due to the loss of Sertoli cells after day 13 of culture. However, Sertoli cells may not be beneficial for survival of SSCs in long-term culture, since it has been reported that Sertoli cell based feeder layers (including SF7 and TM4) cannot efficiently support mouse SSC proliferation in vitro [10]. In addition, the increased population of peritubular cells may provide an alternative source of GDNF during culture [59]. Despite the observed decrease in spermatogonia percentage, we have previously demonstrated the continued presence of SSCs in human long-term testicular cultures after xenotransplantation in the mouse [21]. We also demonstrated that by sorting for ITGA6+ cells before transplantation a sevenfold enrichment in colonizing human SSCs could be obtained during xenotransplantation, confirming that SSCs are still present in long term PTCs [30]. In line with these findings, both Medrano et al. and Baert et al. detected VASA+/UCHL1+ spermatogonia in human testicular cell cultures. More specifically, Baert et al. [29], report single cells or small groups of spermatogonia after 1 month of culture, but decreased numbers after cultures of 2 months, while they detected an increase of VASA−/UCHL1+ in the monolayer, suggesting that UHCL1 by itself does not selectively mark spermatogonial stem cells. In this study, we were able to confirm downregulation of *DDX4* (VASA) and *UTF1* transcripts in long-term cultured ITGA6 + PTCs in line with decrease in percentage spermatogonia. We also detected a slightly increased expression of *UCHL1* in LT-ITGA6 + PTCs, corresponding to expression of the *UCHL1* transcript by some somatic cells originating from the testicular somatic cell population present in our cultured cell fractions. The issue of marker specificity to detect human spermatogonia in vitro has been raised in previous reports and is an ongoing topic of debate [27,28,60,61].

Adding to the overall findings of previous studies, in the current study we describe heterogeneous gene expression patterns over time in culture of ITGA6 + PTC using a panel of 35 established human spermatogonial markers (reviewed in [51]). One would expect all these spermatogonial specific marker genes to show consistent downregulation in long-term cultured ITGA6 + PTCs based on the experience of mentioned studies on human SSC cultures including the current results. While we were able to ascertain the reliability of a large proportion of established in vivo spermatogonial makers that follow this decline in percentage of SSCs, indicating that these markers are suitable for detection of spermatogonia in vitro (*DDX4*, *ELAVL2*, *MAGEA4*, *PASD1*, *FGFR3*, *GFRA1*, *DSG2*, *DMRT1*, *SAGE1*, *UTF1*, *EPCAM*, *TRAPPC6A*, *ZBTB16*, *SALL4*, *SSX3*, *ID4*, *SSX1*, *LIN28A*, *PROM1*, *NANOS2*, *NANOS3*, *SOX3* and *PAX7*), a proportion of spermatogonial markers (*POU2F2, ENO2, SPOCD1, FMR1, ZKSCAN2, GRP125, ITGB1. UCHL1, KIT, EXOSC10* and *CD9*) had expression profiles inconsistent with this decrease and are therefore expected to be expressed by one or more of the other (somatic) cell types present in long-term cultured ITGA6 + PTCs. Follow up studies using single cell RNA-Seq could provide more clarity into the precise expression patterns of different testicular cell types, both in vivo and in vitro. Recently, substantial progress has been made in unravelling the transcriptomic properties of several testicular cell types through single cell RNA-Seq [61,62,63]. Alternatively, Q-PCR and FACS analyses of sorted cell populations within the LT-ITGA6 + PTCs might be another approach to further characterize each of the sorted population for expression of well-known cell type specific markers. Applying these techniques in the context of long-term testicular cell culture may serve to accurately determine stability of in vitro specific spermatogonial markers and should be focused on future research endeavors.

Concurrent with the downregulation of spermatogonial genes, another prominent finding in the present study comes from the gene set enrichment and cell type deconvolution analyses, which revealed increased expression of genes related to EMT. These observations suggest that long-term cultured ITGA6+ sorted fractions might contain a large fraction of mesenchymal cells. This would be in agreement with previous data, where we observed that compact ESC-like colonies formed in PTC cultures, under similar conditions as in current study, resemble mesenchymal cells of testicular origin [34,43] which interestingly do not appear to be the product of EMT, due to the absence of detectable nuclear β-catenin translocation [34]. Moreover, we here showed that ITGA6 + PTCs express *PDGFRA*, *IGFBP5* and *STAR* not concordant with the remaining analyzed (progenitor) Leydig cells markers. Despite the generally accepted idea that adult Leydig cells cannot proliferate in vitro, it was recently shown that PDGFRA is ubiquitously expressed by primary cultured putative human stem Leydig cells, which in turn are suggested to be of testicular mesenchymal origin [64]. In that study, the expression of PDGFRA1 is shown from stem Leydig cells to adult Leydig cells including the perivascular and peritubular cell layers in the human testis. EMT has also been associated with risk of tumor formation, but in a recent study using the same culture procedure no changes in DNA methylation, indicative of tumorigenesis, were observed [35]. In addition, in an allogenic transplantation study using mouse in vitro propagated spermatogonia where the exact same culture medium was used as in our current study, no increased risk of cancer was reported in long-term followed transplanted mice compared to control [65].

On the other hand, the presence of mesenchymal cells in PTCs may also benefit future transplantation purposes as it has been postulated that mesenchymal cells can contribute to the colonization potential of (cultured) human spermatogonial cell fractions. This idea is substantiated by a recent report of Kadam et al. [66], who showed increased efficiency of spermatogonial colonization after co-transplanting mouse SSCs with mouse bone-marrow derived MSCs in a allogeneic transplantation model.

The current culture system for primary testicular cells derived from a testicular biopsy and subsequent enrichment of functional spermatogonia using ITGA6 as a marker would benefit from refinement to increase the percentage of SSCs in this population before it is applied in a clinical setting. To reduce the growth rate of testicular somatic cells and facilitate the proliferation of spermatogonial cells, several aspects of the culture system can be adapted. Mirzapour et al., reported that co-culturing spermatogonial cell fractions with separately isolated Sertoli cells rather than endogenous mixed testicular somatic cells results in increased germ cell colony formation [67]. Alternatively, the use of DMEM-F12 medium over StemPro-34 medium might be beneficial for spermatogonial growth [68]. Other advances have been made with regards to replacing the feeder layer with a cell-free structure, by exploiting hydrogel forms of human testis derived extracellular matrix [69], or three-dimensional agar culture in combination with knockout serum replacement [70]. In addition, changing temperature may be useful to further improve propagation of SSCs in culture, as this was also beneficial in an organotypic testicular organ culture [71]. However, most studies did not culture for long periods of time to determine the long-term effect of these adaptations to the percentages of the various testicular cell types over time in culture or used intact testicular organ fragments in culture rather than isolated cells.

In addition, future directions of the current work may include refinement of the current sorting strategies to either remove or reduce the number of testicular MSCs in long-term cultured PTC fractions through negative cell selection. De Chiara et al. have shown that MSCs derived from testicular biopsies have a CD73+/CD90+/CD105+/CD14−/CD34− phenotype and do not express *VASA* [72]. It would be interesting to determine whether periodic removal of cells expressing this set of markers can increase the efficiency of the human testicular cell culture system and lead to an increased yield of spermatogonial cells and increased colonization potential in a xenotransplantation assay. Additional negative selection procedures for peritubular myoid cells or fibroblasts could serve a similar purpose and should be a subject of future research. Finally, the optimization conditions of cell sorting strategy and cell culture system for clinical use should be performed using prepubertal testicular material.

In conclusion, our data suggest that human ITGA6+ testicular cells contain a decreased spermatogonial gene expression pattern and gain a mesenchymal expression profile in culture. The data also stress that great care should be taken in the choice of marker genes used to identify spermatogonia in vitro. Further refinement and optimization of the human SSC culture system is required in light of potential future fertility restoration therapies that use long-term in vitro propagated spermatogonial stem cells.

## 4. Materials and Methods

### 4.1. Ethics Statement

In the current study, testicular tissues were obtained from prostate cancer patients (*n* = 4, aged 49 (URO0077), 60 (URO0246), 77 (URO0034) and 83 (URO0179)) that underwent a bilateral orchiectomy procedure as part of their cancer treatment. All subjects have given informed oral consent for the use of spare testicular tissue fragments for research purposes. In accordance with Dutch law, no further permission of an ethical committee was required to proceed with the study, since no additional medical interventions were performed to acquire the biological materials.

### 4.2. Patient Samples

Protocols for the procurement, storage, isolation and culturing of mixed primary testicular cells (PTC) from cryopreserved human testicular biopsies were identical to and are described extensively in a previous publication [35]. In short, single cell suspensions were isolated from cryopreserved testicular biopsies using a two-step enzymatic tissue digestion step, followed by overnight differential plating in MEM medium to remove adherent somatic cells from the non-adherent germ cell fraction [73]. The non-adherent cell fraction was transferred to a new dish containing supplemented StemPro-34 SFM medium (10639–011, Life Technologies, Carlsbad, CA, USA) and was cultured at 37 °C in 5% CO_2_. Testicular cells were harvested at six time points during culture, ranging from 0 h up to two months in culture. After harvesting, testicular cell suspensions were sorted for ITGA6+ cells by MACS according to manufacturer’s protocol.

### 4.3. Live Cell Imaging

A cryopreserved aliquot of unsorted PTCs (URO0246, 29 days in culture, passage 3) was thawed and plated in a T25 flask using supplemented StemPro-34 SFM medium and carried over to an incubation chamber calibrated at 37 °C outfitted with a phase contrast microscope. To visualize the behavior of the somatic monolayer, a time-lapse video was recorded over a period of 96 h by sequential photographing with a framerate of two images per minute totaling 11,520 frames (Video S1).

### 4.4. RNA Isolation, Quality Control

Total RNA was extracted from ITGA6+ sorted primary testicular cells using a MagNA Pure LC instrument (Life Technologies, Carlsbad, CA, USA). Samples were scored for RNA integrity using a RNA 6000 Pico kit for the Agilent 2100 Bioanalyzer (Agilent Technologies, Santa Clara, CA, USA) gel-based separation fragment analyzer. Briefly, the ratio of the 18S/28S ribosomal peaks, which is a measure for RNA degradation, was calculated and samples below a cutoff RNA Integrity Number (RIN) value of 7.0 (range 1–10) were considered as being of degraded quality. All samples (24/24) displayed high RNA integrity scores ranging between 7.1 and 9.8 and were deemed suitable for reliable sequence library preparation.

### 4.5. cDNA Amplification

RNA was amplified using the Ovation RNA-Seq System V2 kit (NuGEN Technologies, San Carlos, CA, USA, lot no. 1307479-B) according to manufacturer’s protocol, yielding an average of 7.3 ± 0.9 µg double stranded cDNA per sample using 20 ng RNA input. Even distribution of sequencing reads along the length of each transcript was ensured through combined use of oligo(dT) hexamer and random primers.

### 4.6. Library Preparation

A total of 1 µg amplified cDNA was barcoded with a unique set of standard Illumina TruSeq indexed adapters by ligation and sequenced on an Illumina HiSeq 2000 high-throughput sequencer according to manufacturer’s protocol at the department of Clinical Genetics, Amsterdam UMC Location VUmc in Amsterdam, The Netherlands. Paired-end reads of 2 × 100 bp were trimmed using Trimmomatic (v0.32, https://github.com/timflutre/trimmomatic) [74] (reads ≥ 15 nucleotides passed the filter) and aligned to the hg19 human reference genome using TopHat/Bowtie2 (v2.1.0, https://ccb.jhu.edu/software/tophat/index.shtml) [75]. We recovered 31,370,153 ± 10,903,535 uniquely aligned paired-end sequence reads per sample as generated by samtools (v0.1.19, http://www.htslib.org/) spanning 19,250 ± 1865 known transcripts.

### 4.7. Specification of Testicular Cell Type Marker Genes

Using existent literature available in the PubMed international library database, a comprehensive bibliographical search was performed to identify cell type specific markers expected to be present in testicular cell suspensions based on in vivo expression. This resulted in a list of 106 marker genes that have been reported to be specifically expressed by one of seven distinct cell types: spermatogonia, peritubular myoid cells, endothelial cells, mesenchymal stromal cells, fibroblasts, (progenitor) Leydig cells and Sertoli cells. The total list of gene identifiers and associated literature references can be viewed in Table S1.

### 4.8. Bioinformatics and Statistical Analyses

#### 4.8.1. Count Data Normalization

Counts were obtained using HTSeq (v0.5.4p3, https://htseq.readthedocs.io/en/master/) [76], using the UCSC hg19 genome as the reference genome build for gene annotation. Count data were normalized using edgeR employing a published trimmed mean of M-values method [77,78], resulting in a data matrix containing normalized counts-per-million (CPM) for each gene. Genes expressed (CPM value of ≥ 1) in ≥4/24 samples were selected for further analysis, resulting in a total of 18,380 unique gene identifiers available for downstream analysis. Raw and processed data is available from the GEO online database (accession number GSE158165).

#### 4.8.2. Differential Gene Expression Analysis

Differential expression analyses were performed using a generalized linear model regression approach taking pairing into account. Time in culture was used as a factor and patient as blocking factor. Genes with a log2 fold change of ≥2.0 in both directions and an adjusted (for multiple testing) *p*-value of ≤ 0.01 (FDR) were selected as most likely candidates to be significantly differentially expressed. The number of differentially expressed genes was visualized using vidger (v1.8.0, https://bioconductor.org/packages/release/bioc/html/vidger.html) [79]. For comparisons of transcriptional complexity, a one-way ANOVA was performed followed by pair wise Tukey honest significant differences tests (Tukey HSD) with *p*-values ≤ 0.01 being considered as significantly different.

#### 4.8.3. Gene Set Enrichment Analysis

Gene set enrichment analysis was performed using a correlation adjusted mean rank gene set test (CAMERA) [80], which tests for enrichment of predefined molecular gene set signatures in differential gene expression lists. Curated gene signatures were retrieved from the MSigDB v4.0 online database (http://www.broadinstitute.org/gsea/msigdb). Original gene symbols were mapped to other gene identifiers using biomaRt [81], where necessary.

#### 4.8.4. Cell Mix Decomposition Analysis

Estimations of cell type proportions were generated using Cell Mix [52] version 1.6.2 (http://web.cbio.uct.ac.za/~renaud/CRAN/web/CellMix/), a publically available R package that allows for deconvolution analysis in RNA expression data from heterogeneous biological samples. Default settings were applied to generate the cell type proportion plot and individual marker expression pattern plots. Cell type proportions were tested for significance using a linear regression model per cell type, with average proportions as dependable variable and time point as independent variable (lm () function in R).

## Figures and Tables

**Table 1:** Gene set enrichment analysis of the top 20 gene sets enrichment analyses based on transcriptomic differences between d0 and LT in the ‘C2: Curated gene sets’ category as listed in the MSigDB database, which includes canonical pathways and genetic/chemical perturbations.

Gene Set	# Genes	Direction	*p*-Value	FDR ^1^
OXFORD_RALA_TARGETS_DN	9	Up	1.79 × 10^−6^	0.0163
VERRECCHIA_RESPONSE_TO_TGFB1_C4	13	Up	1.53 × 10^−4^	0.357
VANHARANTA_UTERINE_FIBROID_UP	41	Up	2.29 × 10^−4^	0.357
WEBER_METHYLATED_ICP_IN_FIBROBLAST	17	Down	2.30 × 10^−4^	0.357
PID_INTEGRIN5_PATHWAY	16	Up	2.35 × 10^−4^	0.357
JI_CARCINOGENESIS_BY_KRAS_AND_STK11_DN	15	Up	3.09 × 10^−4^	0.36
CLASPER_LYMPHATIC_VESSELS_DURING_METASTASIS_DN	35	Up	3.16 × 10^−4^	0.36
ONDER_CDH1_TARGETS_3_UP	17	Up	4.35 × 10^−4^	0.385
INGRAM_SHH_TARGETS_DN	59	Up	4.49 × 10^-4^	0.385
WEBER_METHYLATED_ICP_IN_SPERM_DN	11	Down	5.71 × 10^-4^	0.385
SENESE_HDAC1_TARGETS_DN	221	Up	5.78 × 10^−4^	0.385
BROWNE_HCMV_INFECTION_18HR_DN	147	Up	6.47 × 10^−4^	0.385
WEBER_METHYLATED_ICP_IN_SPERM_UP	6	Down	7.13 × 10^−4^	0.385
PID_INTEGRIN1_PATHWAY	61	Up	7.29 × 10^−4^	0.385
ANASTASSIOU_CANCER_MESENCHYMAL_TRANSITION_SIGNATU	60	Up	7.71 × 10^−4^	0.385
KEGG_LYSOSOME	114	Up	8.18 × 10^−4^	0.385
SUZUKI_AMPLIFIED_IN_ORAL_CANCER	11	Up	8.46 × 10^−4^	0.385
REACTOME_GLYCOSPHINGOLIPID_METABOLISM	29	Up	1.00e × 10^−3^	0.388
KEGG_GLYCOSPHINGOLIPID_BIOSYNTHESIS_GANGLIO_SERIES	14	Up	1.02 × 10^−3^	0.388
BROWNE_HCMV_INFECTION_48HR_DN	432	Up	1.06 × 10^−3^	0.388

^1^ FDR significance cutoff set at ≤0.01.

## Supplementary Materials

The following are available online at https://www.mdpi.com/1422-0067/21/21/8269/s1, Figure S1: Differentially expressed gene (DEG) analysis, Figure S2: Gene expression levels of cell type specific markers in ITGA6 + PTCs during culture, Figure S3: K-means clustering analysis, Table S1: List of specific marker genes for seven cell types found in native testis, Video S1: Live cell imaging recording of unsorted PTCs at passage 3.
