# Supplementary material for: ITGA6+ Human Testicular Cell Populations Acquire a Mesenchymal Rather than Germ Cell Transcriptional Signature during Long-Term Culture
Source: Int J Mol Sci. 2020 Nov 4;21(21):8269. doi: 10.3390/ijms21218269 (PMC7672582; doi:10.3390/ijms21218269)
Supplement: Supplementary file 1 [file ijms-21-08269-s001.zip › ijms-961521 supplementary/Table S1. List of specific marker genes for seven cell types found in native testis.docx]

**Table S1.** List of specific marker genes for seven cell types found in native testis. A literature search was performed to establish a minimal marker list of genes specifically expressed in the below mentioned cell types, validated in silico and subsequently used for cell decomposition analysis.

| **Gene** | **Ensemble ID** | **Specificity ^1^** | **Reference (s)** | **Cell Mix ^2^** |
| --- | --- | --- | --- | --- |
| CXCL12 | ENSG00000107562 | END | [[42](#_ENREF_42)] | Y |
| CXCL2 | ENSG00000081041 | END | [[42](#_ENREF_42)] | Y |
| EPAS1 | ENSG00000116016 | END | [[41](#_ENREF_41)] | Y |
| FGFR1 | ENSG00000077782 | END | [[42](#_ENREF_42)] | Y |
| IGFBP2 | ENSG00000115457 | END | [[42](#_ENREF_42)] | Y |
| NOSTRIN | ENSG00000275326 | END | [[41](#_ENREF_41)] | Y |
| PALMD | ENSG00000099260 | END | [[41](#_ENREF_41)] | Y |
| POSTN | ENSG00000133110 | END | [[41](#_ENREF_41)] | Y |
| TGFBR2 | ENSG00000163513 | END | [[41](#_ENREF_41)] | Y |
| VWF | ENSG00000110799 | END | [[41](#_ENREF_41)] | Y |
| APCDD1 | ENSG00000154856 | FIB | [[43](#_ENREF_43)] | Y |
| DKK1 | ENSG00000107984 | FIB | [[43](#_ENREF_43)] | Y |
| MKX | ENSG00000150051 | FIB | [[43](#_ENREF_43)] | Y |
| MMP1 | ENSG00000196611 | FIB | [[43](#_ENREF_43)] | Y |
| MMP10 | ENSG00000166670 | FIB | [[43](#_ENREF_43)] | Y |
| MMP3 | ENSG00000149968 | FIB | [[43](#_ENREF_43)] | Y |
| MOXD1 | ENSG00000079931 | FIB | [[43](#_ENREF_43)] | Y |
| PSG1 | ENSG00000231924 | FIB | [[43](#_ENREF_43)] | Y |
| PSG4 | ENSG00000243137 | FIB | [[43](#_ENREF_43)] | Y |
| S100A4 | ENSG00000196154 | FIB | [[43](#_ENREF_43)] | Y |
| SERPINB2 | ENSG00000197632 | FIB | [[43](#_ENREF_43)] | Y |
| SNAI2 | ENSG00000019549 | FIB | [[43](#_ENREF_43)] | Y |
| STMN2 | ENSG00000104435 | FIB | [[43](#_ENREF_43)] | Y |
| TFAP2A | ENSG00000137203 | FIB | [[43](#_ENREF_43)] | Y |
| CFD | ENSG00000274619 | LEY | [[41](#_ENREF_41)] | Y |
| CYP11A1 | ENSG00000140459 | LEY | [[45](#_ENREF_45)] | Y |
| CYP17A1 | ENSG00000148795 | LEY | [[44](#_ENREF_44)] | Y |
| DLK1 | ENSG00000185559 | LEY | [[41](#_ENREF_41)] | Y |
| HSD17B3 | ENSG00000130948 | LEY | [[44](#_ENREF_44)] | Y |
| HSD3B2 | ENSG00000203859 | LEY | [[44](#_ENREF_44)] | Y |
| IGF1 | ENSG00000017427 | LEY | [[41](#_ENREF_41)] | Y |
| IGF2 | ENSG00000167244 | LEY | [[41](#_ENREF_41)] | Y |
| IGFBP5 | ENSG00000115461 | LEY | [[41](#_ENREF_41)] | N |
| INSL3 | ENSG00000248099 | LEY | [[44](#_ENREF_44)] | Y |
| PDGFRA | ENSG00000134853 | LEY | [[44](#_ENREF_44)] | N |
| STAR | ENSG00000147465 | LEY | [[45](#_ENREF_45)] | N |
| BST2 | ENSG00000130303 | MSC | [[43](#_ENREF_43)] | Y |
| CD200 | ENSG00000091972 | MSC | [[43](#_ENREF_43)] | Y |
| CD44 | ENSG00000026508 | MSC | [[43](#_ENREF_43)] | Y |
| ENG | ENSG00000106991 | MSC | [[43](#_ENREF_43)] | Y |
| FN1 | ENSG00000115414 | MSC | [[43](#_ENREF_43)] | Y |
| FZD1 | ENSG00000157240 | MSC | [[43](#_ENREF_43)] | Y |
| FZD4 | ENSG00000174804 | MSC | [[43](#_ENREF_43)] | Y |
| IL6ST | ENSG00000134352 | MSC | [[43](#_ENREF_43)] | Y |
| ITGA11 | ENSG00000137809 | MSC | [[43](#_ENREF_43)] | Y |
| JAG1 | ENSG00000101384 | MSC | [[43](#_ENREF_43)] | Y |
| LTBP1 | ENSG00000049323 | MSC | [[43](#_ENREF_43)] | Y |
| MCAM | ENSG00000076706 | MSC | [[43](#_ENREF_43)] | Y |
| NFIB | ENSG00000147862 | MSC | [[43](#_ENREF_43)] | Y |
| NGFR | ENSG00000064300 | MSC | [[43](#_ENREF_43)] | Y |
| NOTCH3 | ENSG00000074181 | MSC | [[43](#_ENREF_43)] | Y |
| NT5E | ENSG00000135318 | MSC | [[47](#_ENREF_47)] | Y |
| PDGFRB | ENSG00000113721 | MSC | [[43](#_ENREF_43)] | Y |
| PODXL | ENSG00000128567 | MSC | [[46](#_ENREF_46)] | Y |
| SEC13 | ENSG00000157020 | MSC | [[43](#_ENREF_43)] | Y |
| SUSD2 | ENSG00000099994 | MSC | [[46](#_ENREF_46)] | Y |
| TM4SF1 | ENSG00000169908 | MSC | [[43](#_ENREF_43)] | Y |
| VCAM1 | ENSG00000162692 | MSC | [[43](#_ENREF_43)] | Y |
| VCAN | ENSG00000038427 | MSC | [[43](#_ENREF_43)] | Y |
| ACTA2 | ENSG00000107796 | PMC | [[48](#_ENREF_48)] | Y |
| ICAM1 | ENSG00000090339 | PMC | [[49](#_ENREF_49)] | Y |
| MYH11 | ENSG00000133392 | PMC | [[41](#_ENREF_41)] | Y |
| MYL9 | ENSG00000101335 | PMC | [[41](#_ENREF_41)] | Y |
| TPM1 | ENSG00000140416 | PMC | [[41](#_ENREF_41)] | Y |
| TPM2 | ENSG00000198467 | PMC | [[41](#_ENREF_41)] | Y |
| TPM4 | ENSG00000167460 | PMC | [[41](#_ENREF_41)] | Y |
| AR | ENSG00000169083 | SER | [[25](#_ENREF_25)] | Y |
| FGF2 | ENSG00000138685 | SER | [[25](#_ENREF_25)] | Y |
| GATA4 | ENSG00000136574 | SER | [[25](#_ENREF_25)] | Y |
| SOX9 | ENSG00000125398 | SER | [[50](#_ENREF_50)] | Y |
| WT1 | ENSG00000184937 | SER | [[25](#_ENREF_25)] | Y |
| CD9 | ENSG00000010278 | SPG | [[51](#_ENREF_51)] | N |
| CHEK2 | ENSG00000183765 | SPG | [[51](#_ENREF_51)] | N |
| DDX4 | ENSG00000152670 | SPG | [[63](#_ENREF_63)] | Y |
| DMRT1 | ENSG00000137090 | SPG | [[51](#_ENREF_51)] | Y |
| DSG2 | ENSG00000046604 | SPG | [[51](#_ENREF_51)] | Y |
| ELAVL2 | ENSG00000107105 | SPG | [[51](#_ENREF_51)] | Y |
| ENO2 | ENSG00000111674 | SPG | [[51](#_ENREF_51)] | N |
| EPCAM | ENSG00000119888 | SPG | [[51](#_ENREF_51)] | Y |
| EXOSC10 | ENSG00000171824 | SPG | [[51](#_ENREF_51)] | N |
| FGFR3 | ENSG00000068078 | SPG | [[51](#_ENREF_51)] | Y |
| FMR1 | ENSG00000102081 | SPG | [[51](#_ENREF_51)] | N |
| GFRA1 | ENSG00000151892 | SPG | [[51](#_ENREF_51)] | Y |
| GPR125 | ENSG00000152990 | SPG | [[51](#_ENREF_51)] | N |
| ID4 | ENSG00000172201 | SPG | [[51](#_ENREF_51)] | Y |
| ITGB1 | ENSG00000150093 | SPG | [[51](#_ENREF_51)] | N |
| KIT | ENSG00000157404 | SPG | [[51](#_ENREF_51)] | N |
| LIN28A | ENSG00000131914 | SPG | [[51](#_ENREF_51)] | Y |
| MAGEA4 | ENSG00000147381 | SPG | [[51](#_ENREF_51)] | Y |
| NANOS2 | ENSG00000188425 | SPG | [[51](#_ENREF_51)] | Y |
| NANOS3 | ENSG00000187556 | SPG | [[51](#_ENREF_51)] | Y |
| PASD1 | ENSG00000166049 | SPG | [[51](#_ENREF_51)] | Y |
| PAX7 | ENSG00000009709 | SPG | [[51](#_ENREF_51)] | Y |
| POU2F2 | ENSG00000028277 | SPG | [[51](#_ENREF_51)] | N |
| PROM1 | ENSG00000007062 | SPG | [[51](#_ENREF_51)] | Y |
| SAGE1 | ENSG00000181433 | SPG | [[51](#_ENREF_51)] | Y |
| SALL4 | ENSG00000101115 | SPG | [[51](#_ENREF_51)] | Y |
| SOX3 | ENSG00000134595 | SPG | [[51](#_ENREF_51)] | Y |
| SPOCD1 | ENSG00000134668 | SPG | [[51](#_ENREF_51)] | N |
| SSX1 | ENSG00000126752 | SPG | [[51](#_ENREF_51)] | Y |
| SSX3 | ENSG00000165584 | SPG | [[51](#_ENREF_51)] | Y |
| TRAPPC6A | ENSG00000007255 | SPG | [[51](#_ENREF_51)] | Y |
| UCHL1 | ENSG00000154277 | SPG | [[51](#_ENREF_51)] | N |
| UTF1 | ENSG00000171794 | SPG | [[51](#_ENREF_51)] | Y |
| ZBTB16 | ENSG00000109906 | SPG | [[51](#_ENREF_51)] | Y |
| ZKSCAN2 | ENSG00000155592 | SPG | [[51](#_ENREF_51)] | N |

^1^ Abbreviation of the cell type corresponding to the marker gene. END, endothelial cells; FIB, fibroblasts; LEY, (progenitor) Leydig cells; MSC, mesenchymal stromal cells; PMC, peritubular myoid cells; SER, Sertoli cells; SPG, spermatogonia. ^2^ Indication whether the gene was included in the cell type deconvolution analysis (Yes or No).
