# Supplementary figures and images for: ITGA6+ Human Testicular Cell Populations Acquire a Mesenchymal Rather than Germ Cell Transcriptional Signature during Long-Term Culture
Source: Int J Mol Sci. 2020 Nov 4;21(21):8269. doi: 10.3390/ijms21218269 (PMC7672582; doi:10.3390/ijms21218269)

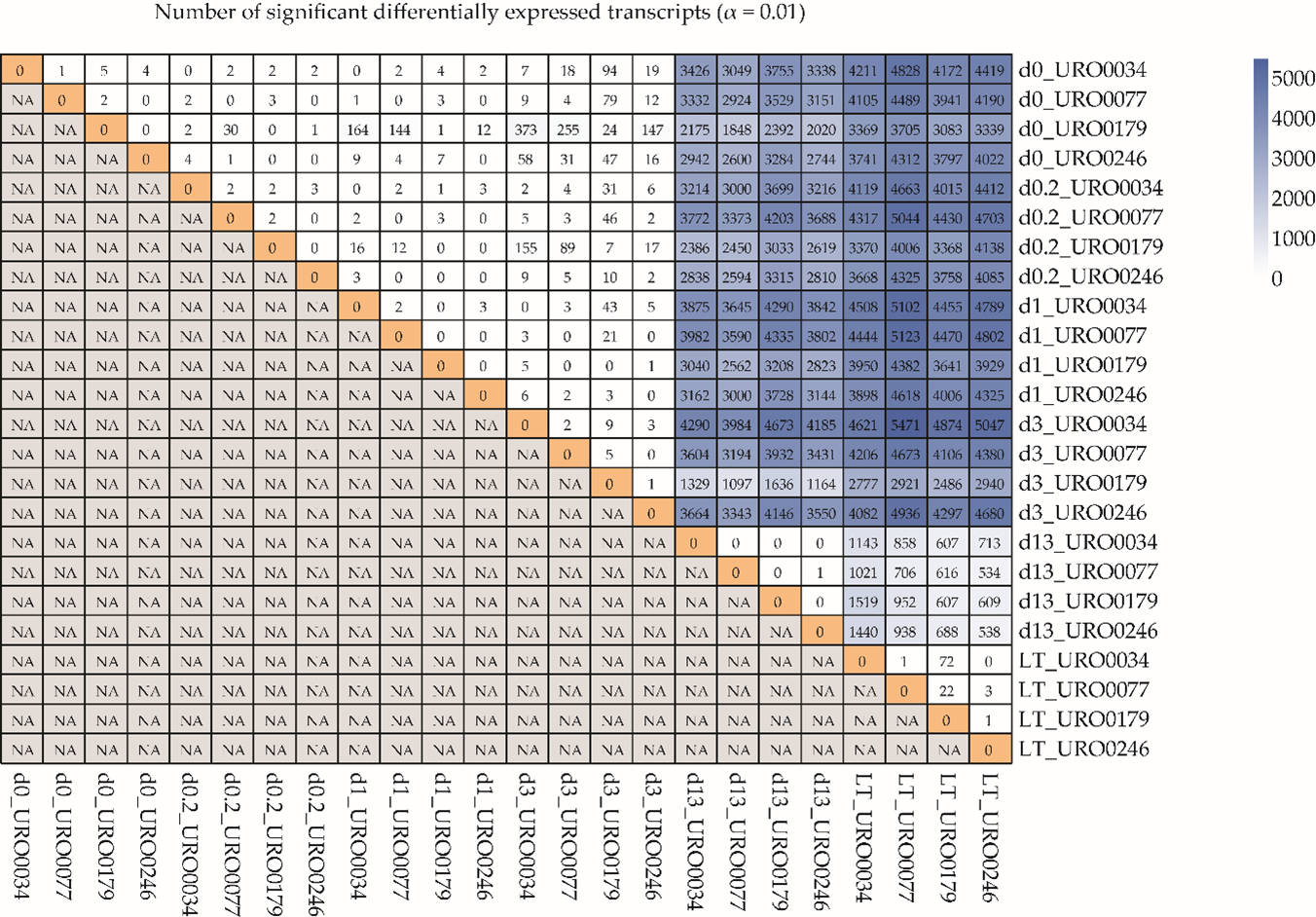

Supplement: Supplementary file 1 [file ijms-21-08269-s001.zip › ijms-961521 supplementary/Figure S1. Differentially expressed gene (DEG) analysis.png]

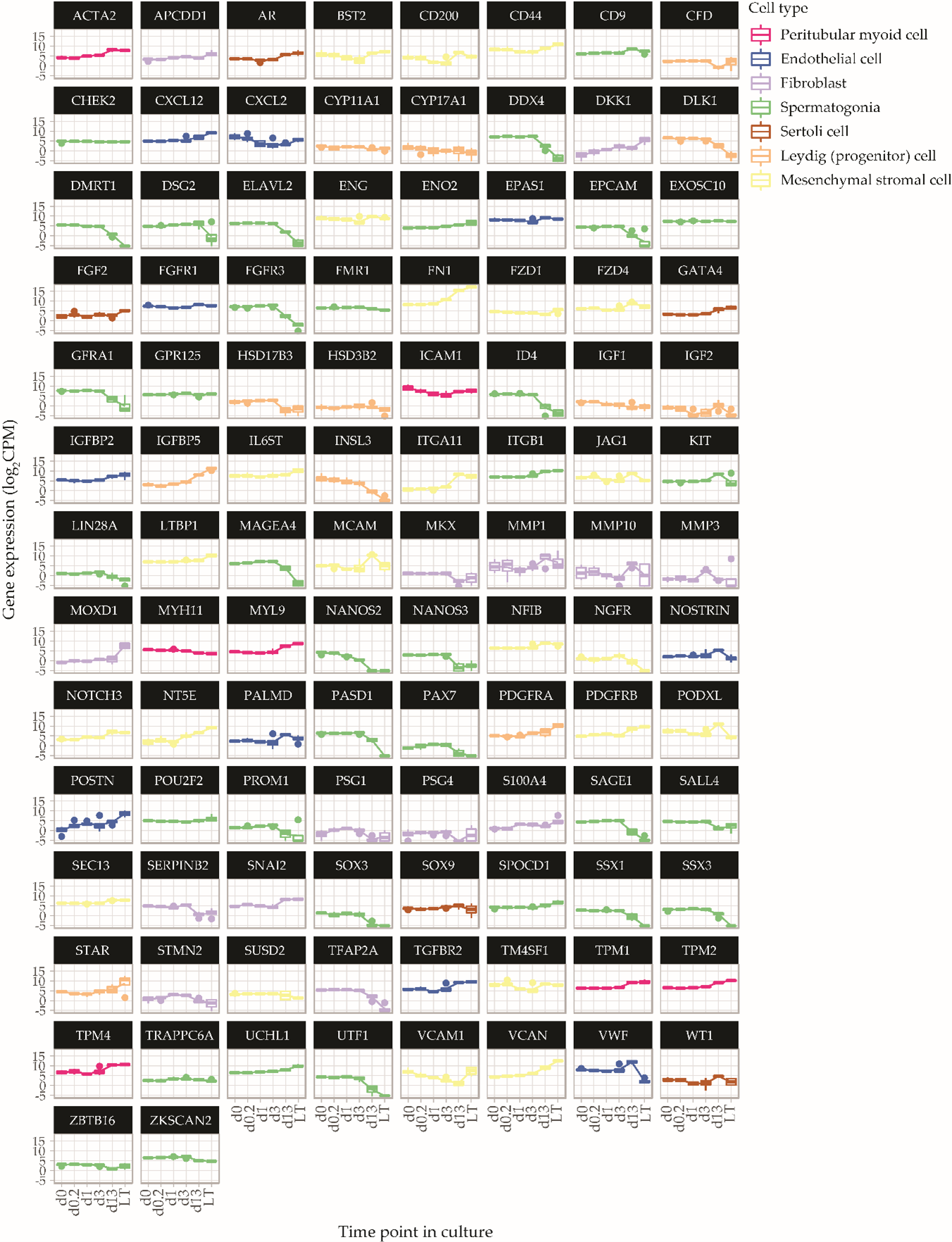

Supplement: Supplementary file 1 [file ijms-21-08269-s001.zip › ijms-961521 supplementary/Figure S2. Gene expression levels of cell type specific markers in ITGA6 + PTCs during culture.png]

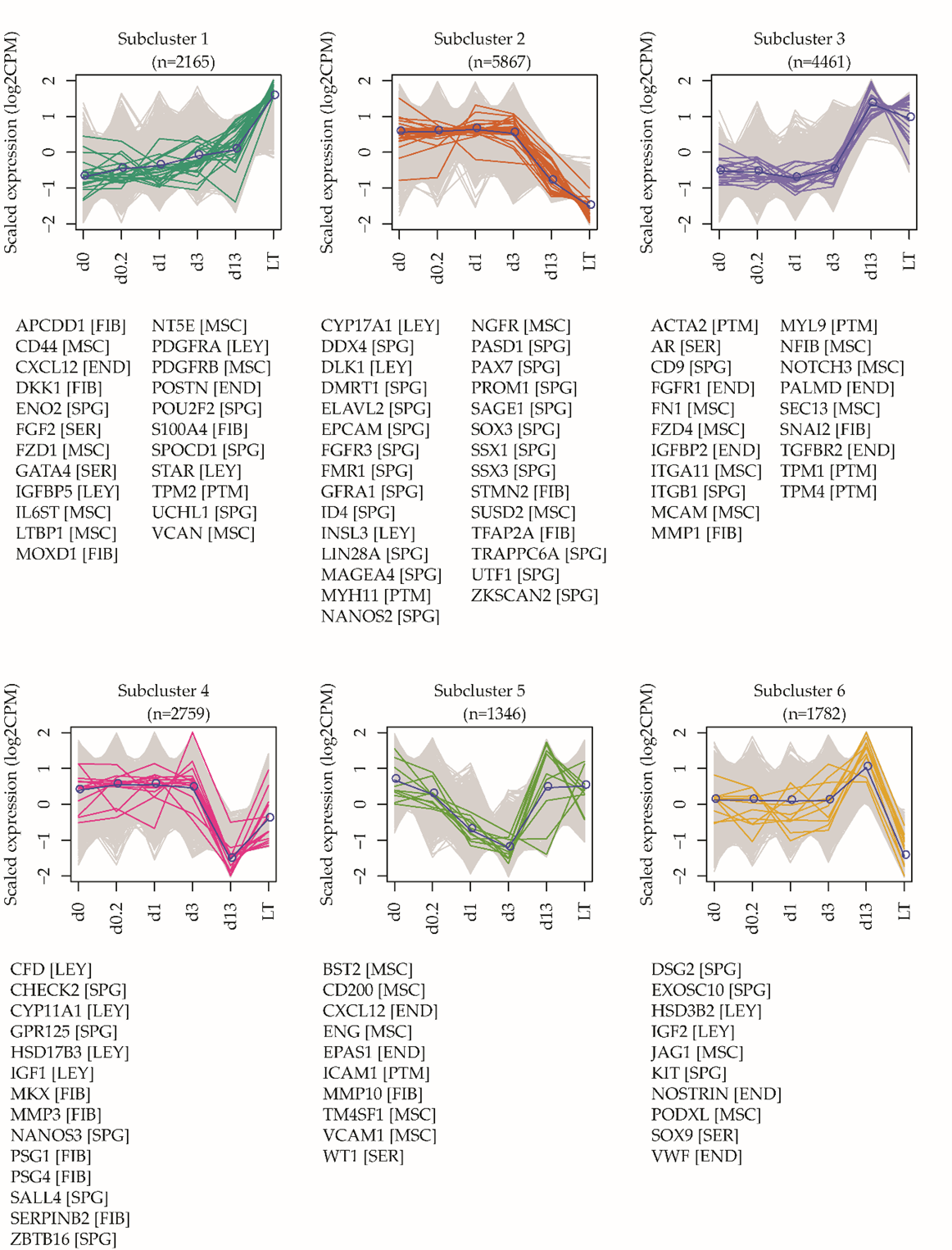

Supplement: Supplementary file 1 [file ijms-21-08269-s001.zip › ijms-961521 supplementary/Figure S3. K-means clustering analysis.png]
